# Improving the Sensitivity of Task-Based Multi-Echo Functional Magnetic Resonance Imaging via *T*_2_* Mapping Using Synthetic Data-Driven Deep Learning

**DOI:** 10.3390/brainsci14080828

**Published:** 2024-08-17

**Authors:** Yinghe Zhao, Qinqin Yang, Shiting Qian, Jiyang Dong, Shuhui Cai, Zhong Chen, Congbo Cai

**Affiliations:** Department of Electronic Science, Xiamen University, Xiamen 361005, China; 33320211150273@stu.xmu.edu.cn (Y.Z.); qqyang@stu.xmu.edu.cn (Q.Y.); 33320211150265@stu.xmu.edu.cn (S.Q.); jydong@xmu.edu.cn (J.D.); shcai@xmu.edu.cn (S.C.); chenz@xmu.edu.cn (Z.C.)

**Keywords:** multi-echo fMRI, synthetic data-driven deep learning, *T*_2_* mapping, BOLD sensitivity

## Abstract

(1) Background: Functional magnetic resonance imaging (fMRI) utilizing multi-echo gradient echo-planar imaging (ME-GE-EPI) has demonstrated higher sensitivity and stability compared to utilizing single-echo gradient echo-planar imaging (SE-GE-EPI). The direct derivation of *T*_2_* maps from fitting multi-echo data enables accurate recording of dynamic functional changes in the brain, exhibiting higher sensitivity than echo combination maps. However, the widely employed voxel-wise log-linear fitting is susceptible to inevitable noise accumulation during image acquisition. (2) Methods: This work introduced a synthetic data-driven deep learning (SD-DL) method to obtain *T*_2_* maps for multi-echo (ME) fMRI analysis. (3) Results: The experimental results showed the efficient enhancement of the temporal signal-to-noise ratio (tSNR), improved task-based blood oxygen level-dependent (BOLD) percentage signal change, and enhanced performance in multi-echo independent component analysis (MEICA) using the proposed method. (4) Conclusion: *T*_2_* maps derived from ME-fMRI data using the proposed SD-DL method exhibit enhanced BOLD sensitivity in comparison to *T*_2_* maps derived from the LLF method.

## 1. Introduction

The gradient echo-planar imaging (GE-EPI) technique [1,2,3], known for its high sensitivity to *T*_2_* variation indicative of blood oxygen level-dependent (BOLD) change, has been widely utilized in functional magnetic resonance imaging (fMRI). It is crucial to achieve a balance between signal-to-noise ratio and sensitivity to the BOLD effect across the entire brain range in collecting *T*_2_*-weighted images in fMRI. Typically, the optimal BOLD effect is achieved when the echo time (TE) of imaging sequence equals the *T*_2_* value [4]. However, finding a global optimal TE value for the best *T*_2_* weighting is challenging due to individual and regional brain differences. In these cases, using multi-echo gradient echo-planar imaging (ME-GE-EPI) with different TEs presents a suitable choice, albeit at the expense of spatio-temporal resolution. Multi-echo fMRI (ME-fMRI), typically acquired using ME-GE-EPI, has proven effective in optimizing BOLD contrast by amalgamating time courses of various TEs using optimized weighting schemes [4,5,6], leading to enhanced BOLD-contrast sensitivity and decreased artifacts.

Furthermore, fMRI is susceptible to physical and physiological interferences, such as head motion [7,8,9], scanner instability [10], respiration [11,12], heartbeat [13,14], cerebrospinal fluid [15], and the pooling of blood in veins [16], which can introduce non-BOLD interference signals. ME-fMRI can eliminate non-BOLD noise by leveraging the TE dependency of the BOLD signal. The performance of ME-fMRI can be further improved with multi-echo independent component analysis (MEICA) [17]. By identifying the reliance of independent components on TE, MEICA can differentiate between BOLD and non-BOLD signals. It has been demonstrated that MEICA efficiently reduces the interferences generated by head movements [18,19]. Additionally, MEICA does not affect the analysis of the default mode network [19], and it improves the identification of the brain network in resting-state fMRI of humans at 3T [17] and rats at 11.7T [20].

The *T*_2_* map estimated by ME-fMRI can improve the comparability of fMRI studies across multiple scanners and imaging centers. Compared to echo combination methods, the *T*_2_* map exhibits higher BOLD sensitivity [21]. Moreover, the *T*_2_* map can be incorporated into the weighting coefficient calculation to determine the echo combination strategy [4,17,18] and to aid in anatomical–functional registration [22].

In ME-fMRI, traditional voxel-wise fitting methods (e.g., log-linear fitting, LLF) are usually used to obtain *T*_2_^*^ according to the exponential decay model [23,24,25,26,27]. However, the noise acquired during magnetic resonance signal acquisition cannot be accurately estimated or removed via voxel-by-voxel fitting under limited echoes. Furthermore, numerical instability during the fitting may magnify noise [24]. The abovementioned issues result in poor-quality *T*_2_* maps, leading to compromised performance of ME-fMRI in brain functional analysis. Consequently, researchers still utilize *T*_2_*-weighted images [6,24,28,29,30] rather than quantitative *T*_2_* maps in fMRI analysis.

In recent years, fMRI analysis has benefited from the emergence of deep learning. In delving into the application of supervised deep learning in the medical domain, a pivotal challenge lies in its heavy reliance on abundant sample–label pairs. Theoretically, an expansion of the training sample size could amplify the model’s generalization capacity, which is paramount for refining diagnostic precision and predictive accuracy. Nevertheless, in the realm of medical practice, the pursuit of large-scale, precisely annotated sample–label pairs is fraught with obstacles. The vast variability in MRI experimental protocols and the intricacies of extracting representative samples from complex real-world settings conspire to make the scarcity of samples an inherent challenge in medical data analysis. This limitation impedes the effective utilization of traditional supervised learning approaches during data preprocessing. To overcome this challenge, the use of synthetic data-driven deep learning techniques has emerged as a promising avenue of research. These techniques could generate a substantial number of simulated samples, thereby compensating for the scarcity of real-world data. And synthetic data-driven training has been increasingly utilized in deep learning-based MRI reconstruction [31,32,33].

In this work, we propose a synthetic data-driven deep learning method (SD-DL) for ME-fMRI *T*_2_* mapping and compare the effects of LLF-fitted and SD-DL-fitted *T*_2_* maps on BOLD sensitivity. We generated synthetic training data and corresponding labels using the MR signal model and two-dimensional virtual parametric maps. Then, the model used in SD-DL was subjected to supervised training using the synthetic data and corresponding labels to remove non-BOLD noise and fit the MR parameters. Unlike voxel-by-voxel fitting, SD-DL performs slice-by-slice fitting. On the one hand, a large amount of prior information about the brain *T*_2_* map can be learned through synthetic data. On the other hand, spatial correlation can be efficiently utilized to obtain accurate *T*_2_* maps. The proposed SD-DL method is validated using a public dataset and a case including in-house data.

## 2. Materials and Methods

### 2.1. Multi-Echo fMRI Signal Model

The fMRI signal obeys the mono-exponential decay model, in which the acquired fMRI signal of a voxel can be described as:

(1) $$\begin{array}{c} S\left(t\right)=S_{0}\cdot e^{-\frac{t}{T_{2}^{*}}}+\varepsilon =S_{0}\cdot e^{-t\cdot R_{2}^{*}}+\varepsilon \approx S_{0}\cdot e^{-t\cdot R_{2}^{*}} \end{array}$$

where *S*(*t*) is the acquired fMRI signal at time *t*, *S*_0_ is the non-BOLD signal related to proton density, *T*_2_* is the transverse relaxation time, *R*_2_* is the inverse of *T*_2_*, and ε is noise.

In ME-fMRI, *T*_2_* and *S*_0_ can be solved by LLF:

(2) $$\begin{array}{c} \left[\begin{array}{c} \log\left(S_{0}\right)\\ R_{2}^{*} \end{array}\right]=pinv\left(\left[\begin{array}{c} 1,-TE_{1}\\ 1,-TE_{2}\\ \vdots \\ 1,-TE_{n} \end{array}\right]\right)\times \left[\begin{array}{c} \log\left(S\left(TE_{1}\right)\right)\\ \log\left(S\left(TE_{2}\right)\right)\\ \vdots \\ \log\left(S\left(TE_{n}\right)\right) \end{array}\right] \end{array}$$

where pinv(·) is a pseudo-inverse operator.

Meanwhile, the voxel-wise linear combination of each echo can be calculated. In this study, we use the *T*_2_*-weighted combination [4,17] to investigate the differences in performance between LLF and the proposed SD-DL in *T*_2_* fitting. The *T*_2_*-weighted combination is referred to as echo combination (EC) in the following. The weighting factor of EC is as follows:

(3) $$w_{n}^{EC}=\frac{TE_{n}^{*}\exp(-\frac{TE_{n}}{{\hat{T}}_{2}^{*}})}{\sum_{i=1}^{N}TE_{i}^{*}\exp(-\frac{TE_{i}}{{\hat{T}}_{2}^{*}})}$$

where *n* indicates the *n*th echo, *N* refers to the total number of echoes, and ${\hat{T}}_{2}^{*}$ refers to the voxel-wise estimation of *T*_2_*.

### 2.2. Data Processing Pipeline

**Figure 1:**
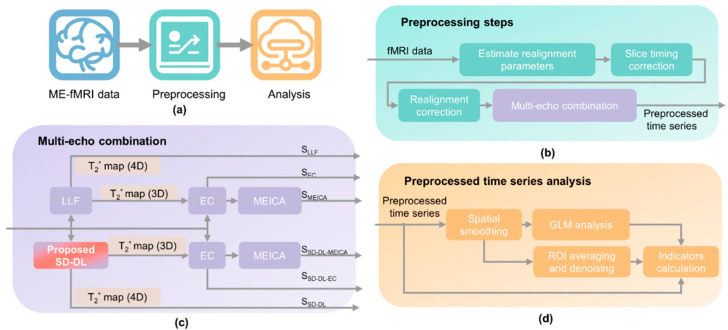
Overview of ME-fMRI processing pipeline. (**a**) Overview of ME-fMRI data preprocessing. (**b**) Preprocessing step. (**c**) Six time series calculated after preprocessing for comparison: S_LLF_ indicates 4-dimensional *T*_2_* map estimated by log-linear fitting (LLF), S_EC_ indicates echo combination (EC) time series, S_MEICA_ indicates MEICA-denoised EC time series, S_SD-DL_ indicates 4-dimensional *T*_2_* map estimated by SD-DL, S_SD-DL-EC_ indicates SD-DL-EC time series, and S_SD-DL-MEICA_ indicates MEICA-denoised SD-DL-EC time series. (**d**) Analysis pipeline.

Figure 1 shows the processing pipeline of ME-fMRI data. The preprocessing was carried out on Matlab (R2021, MathWorks, Natick, MA, USA) and through using the Statistical Parametric Mapping (SPM) 12 toolbox [34]. The flowchart in Figure 1a gives an overview of ME-fMRI data preprocessing. The steps were as follows: In the data preprocessing stage (Figure 1b), realignment parameters were first calculated using ME-fMRI data, and then, slice timing correction and realignment correction were carried out. Subsequently, six time series were calculated based on the realignment-corrected time series (Figure 1c). Two four-dimensional (4D) *T*_2_* fMRI time series were fitted by LLF and SD-DL, respectively. EC and MEICA-denoised EC time series were calculated with *T*_2_* estimated by LLF, and SD-DL-EC and MEICA-denoised SD-DL-EC time series were calculated with *T*_2_* estimated by SD-DL. Finally, indicator calculation and statistical analysis were conducted on six different time series to compare LLF with SD-DL in reflecting brain function (Figure 1d). After the multi-echo combination was completed, spatial smoothing (Gaussian kernel with 7 mm full-width at the half of the maximum, FWHM) and first-level Generalized Linear Model (GLM) analysis were applied to all the time series to extract the BOLD signals. For the rest of this paper, the *T*_2_* fMRI time series fitted by LLF and SD-DL are referred to as S_LLF_ and S_SD-DL_, respectively. The EC and MEICA-denoised EC time series are referred to as S_EC_ and S_MEICA_, respectively. The SD-DL-EC and MEICA-denoised SD-DL-EC time series are referred to as S_SD-DL-EC_ and S_SD-DL-MEICA_, respectively.

### 2.3. T_2_* Mapping and Multi-Echo Combination

**Figure 2:**
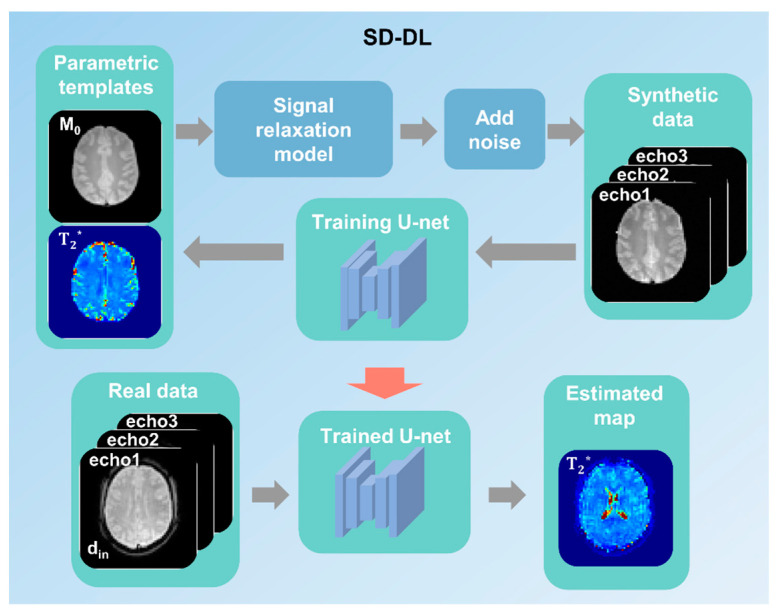
A schematic demonstration of the synthetic data-driven multi-echo deep learning (SD-DL) framework. Parametric templates were transformed from a public multi-contrast image database. Synthetic data were generated from parametric templates through using the fMRI signal relaxation model.

Supervised learning was used for SD-DL *T*_2_* mapping with a 4-layer U-net model. The overall framework is shown in Figure 2. First, two-dimensional (2D) parametric templates (*M_0_* and *T*_2_*) were produced from the public database IXI (https://brain-development.org/ixi-dataset/ (accessed on 15 August 2024)) by the method described by Yang [32] and were down-sampled to a matrix of 64 × 64 to match the matrix size of real data. Then, synthetic multi-echo MR data with TE equal to the real data were generated through Equation (1). Randomized Rician noise was added to the signal magnitude of synthetic data with a standard deviation of 0–5% to mimic a real situation. Additionally, data augmentation was carried out to augment the number of training samples (i.e., random rotation and flip [35]).

The synthetic data were fed into the U-net for model training, and the corresponding parametric templates were used as training labels. Subsequently, the *T*_2_* maps of real data were estimated on the trained U-net model. Overall, 5400 synthesized samples were randomly divided into a training set (4500 samples) and a validation set (900 samples). The training and validation sets were derived from different subjects to avoid data leakage. The random cropping technique was used during model training to improve the model’s generalization ability.

For comparison, S_MEICA_ and S_EC_ were calculated with the Tedana toolbox [17,36,37,38]. To calculate S_SD-DL-EC_, the preprocessed time series was firstly averaged over the time dimension, and then the 3D *T*_2_* maps were subjected to slice-by-slice estimation with SD-DL; secondly, the 3D SD-DL-estimated *T*_2_* maps were used voxel by voxel as ${\hat{T}}_{2}^{*}$ in Equation (3) to calculate the weighting factor of S_SD-DL-EC_.

For SD-DL, the hyperparameters of the model were set as follows: batch size = 16; random crop size = 32 × 32; optimizer = Adam; and learning rate = $10^{-4}$. Upon reaching the lowest loss on the test set, the model was considered fully trained. Synthetic data generation, model training, and pipeline testing were carried out using Python 3.6 in the Pycharm library. All processes were performed with an NVIDIA GeForce RTX 2080Ti GPU.

### 2.4. Data Collection

The study was approved by the institutional research ethics committees, and written informed consent was obtained from all individual participants before the experiments. We conducted experiments based on the real-time multi-echo fMRI (rt-ME-fMRI) dataset [39]. The rt-ME-fMRI dataset, which contains data from 28 participants (20 male and 8 female; mean age = 24.9 (years), SD = 4.7), was accessed via a data use agreement. After excluding corrupted data, the fMRI and physiological data of 27 participants were used in the study. Task-based data were collected using an ON/OFF design, starting from OFF, and each condition lasted for 10 volumes. For each participant, three-echo fMRI data and cardiac and respiratory fluctuations were used for analysis. Cardiac and respiratory fluctuations were sampled at 500 Hz. In GLM analysis, a total of 20 regression variable parameters were used, including 6 realignment parameters and 14 RETROICOR [14] regressors. The RETROICOR regressors were estimated with the TAPAS PhysIO toolbox [40] (both cardiac and respiratory to the 2nd order, excluding interaction regressors). We also collected in-house ME-fMRI data from a healthy volunteer (male, 27-year-old) to further evaluate the performance of the proposed method.

### 2.5. MRI Protocol

Furthermore, rt-ME-fMRI dataset images were acquired using a 3T Philips Achieva scanner with a 32-channel head coil [39]. For each volunteer, anatomical images were acquired using a 3D gradient echo sequence, while fMRI scans were conducted using a multi-echo EPI sequence. The parameters used were as follows:

Anatomical MRI: time of repetition (TR) = 8.2 ms; TE = 3.75 ms; pulse flip angle = 8°; field of view (FOV) = 240 × 240 × 180 mm^3^; and resolution = 1 × 1 × 1 mm^3^. Functional MRI: TR = 2000 ms; TEs = 14, 28, 42 ms (3 echoes); number of volumes = 210 (excluding 5 dummy volumes discarded by the scanner); total scan time = 7 min (excluding 5 dummy volumes); pulse flip angle = 90°; FOV = 224 × 224 × 119 mm^3^; resolution = 3.5 × 3.5 × 3.5 mm^3^; in-plane matrix size = 64 × 64; number of slices = 34; interslice gap = 0 mm; slice orientation = oblique; slice order/direction = sequential/ascending; and SENSE acceleration factor = 2.5.

In-house fMRI data were acquired using a whole-body MRI scanner at 3T (Prisma; Siemens Healthcare, Erlangen, Germany) with a 20-channel head coil. The acquisition parameters were as follows: resolution = 3.5 × 3.5 × 3.5 mm^3^; TR = 2500 ms; TEs = 17, 42, 67 ms (3 echoes); slice number = 32; GRAPPA acceleration factor = 2; and number of volumes = 120 (task-based) and 160 (resting-state). The other imaging parameters were the same as those used for the rt-ME-fMRI dataset. A conventional block-design visual stimulation task with “ON-OFF” blocks (30 s “checkerboard”, followed by a 30 s black field) was performed, in which the “ON” block was used to introduce visual stress. The participant was asked to lay down in a relaxed position in the MRI scanner to minimize noise signals. For comparison, a conventional 2D multi-echo gradient echo sequence was also acquired as the ground truth (GT) of resting state for in-house ME-fMRI data (TR = 3500 ms; TEs = 10, 23, 36, 50, 57.5, and 65 ms; and resolution = 3.5 × 3.5 × 3.5 mm^3^).

### 2.6. Evaluation Indicators

To evaluate our results, mean square error (MSE) analysis was performed on simulation samples under different levels of Rician noise. A temporal signal-to-noise ratio (tSNR) map was calculated by dividing the voxel-wise time series mean by the voxel-wise time series standard deviation (SD) following the elimination of the linear and quadratic trends with the fMRwhy toolbox [41]. To evaluate the sensitivity of S_LLF_ and S_SD-DL_, activation maps (T-value maps) and BOLD response curves of the maximum T-value voxel were illustrated. To evaluate the rt-ME-fMRI data, in addition to the tSNR, the mean of percentage signal change (PSC) [42], T-value, and functional contrast (FC) were illustrated in a target region of interest (ROI).

PSC was used to quantify BOLD sensitivity, which was computed as follows:

(4) $$\begin{array}{c} PSC=\frac{100\times SF\times {\hat{\beta }}_{condition}\;}{{\hat{\beta }}_{constant}} \end{array}$$

where ${\hat{\beta }}_{condition}$ and ${\hat{\beta }}_{constant}$ are parameter maps of condition and constant from GLM analysis, respectively, and the scaling factor (SF) is the maximum value of a reference trial at the resolution of the super-sampled design matrix. The average T-values were used as a proxy for the contrast-to-noise ratio and computed within significantly active voxels inside target ROIs. FC was used as a proxy for signal sensitivity, and for the preprocessed time series, the removal of linear and quadratic trends was first performed, followed by spatial smoothing and GLM analysis and, lastly, spatial averaging within the target ROIs. Subsequently, the mean of the time series in the “ON” condition was subtracted from the mean of the time series in the “OFF” condition to obtain FC.

To avoid scan-specific bias, a binary mask with *p* < 0.001, i.e., the union of family-wise error (UFWE) regions of six time series, was used for statistical analysis. This binary mask will hereafter be referred to as the UFWE ROI. A two-tailed paired *t*-test was applied to the results, and Cohen’s *d* was calculated to identify significant differences, as well as the effect size, between different pipelines.

## 3. Results

### 3.1. Results of Simulation Experiments

**Figure 3:**
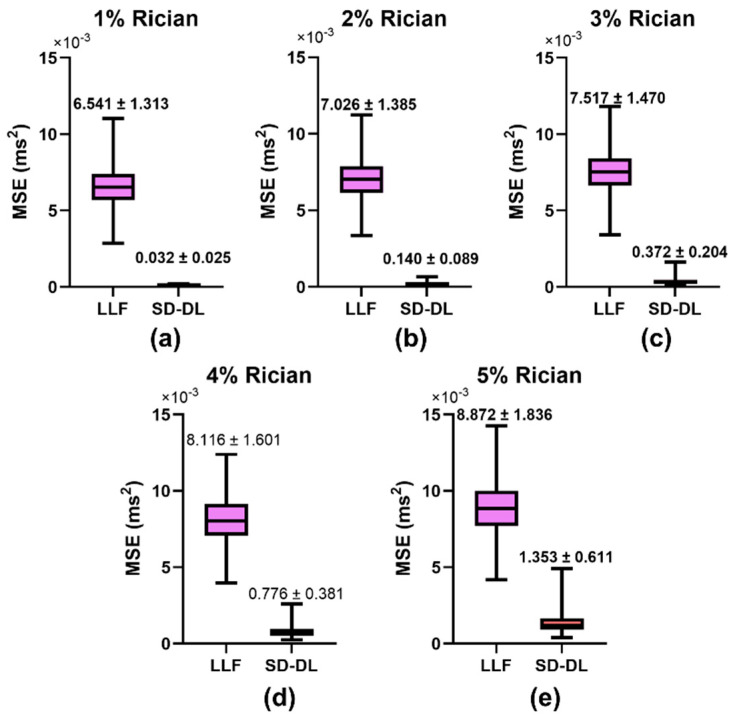
Comparison of mean square error (MSE) between LLF-fitted *T*_2_*, SD-DL-fitted *T*_2_*, and *T*_2_* labels across synthetic samples that had 5 different levels of Rician noise added to them. Rician noise level: (**a**) 1% (**b**) 2% (**c**) 3% (**d**) 4%, and (**e**) 5% standard deviation. Purple color indicates results using the LLF and orange-red color indicates results using the SD-DL.

Figure 3 illustrates the comparative results of the MSEs for the LLF method and SD-DL method across five distinct levels of Rician noise contamination. In the training phase of the SD-DL neural network, a stochastic Rician noise intensity varying from 0% to 5% standard deviation was introduced into the synthesized dataset. For the MSE evaluation, synthetic samples embodying Rician noise at five discrete levels, ranging from 1% to 5%, were produced. The progression depicted in Figure 3a–e revealed a shared trend wherein the MSE values for both SD-DL and LLF escalated in conjunction with the increase in noise magnitude in the synthetic data. Notably, the average rise in MSE for LLF surpassed that of SD-DL. Furthermore, SD-DL consistently achieved significantly reduced MSEs relative to LLF across all examined noise intensity tiers, highlighting its superior denoising efficiency.

### 3.2. Results of Visual Stimulation Task Data

**Figure 4:**
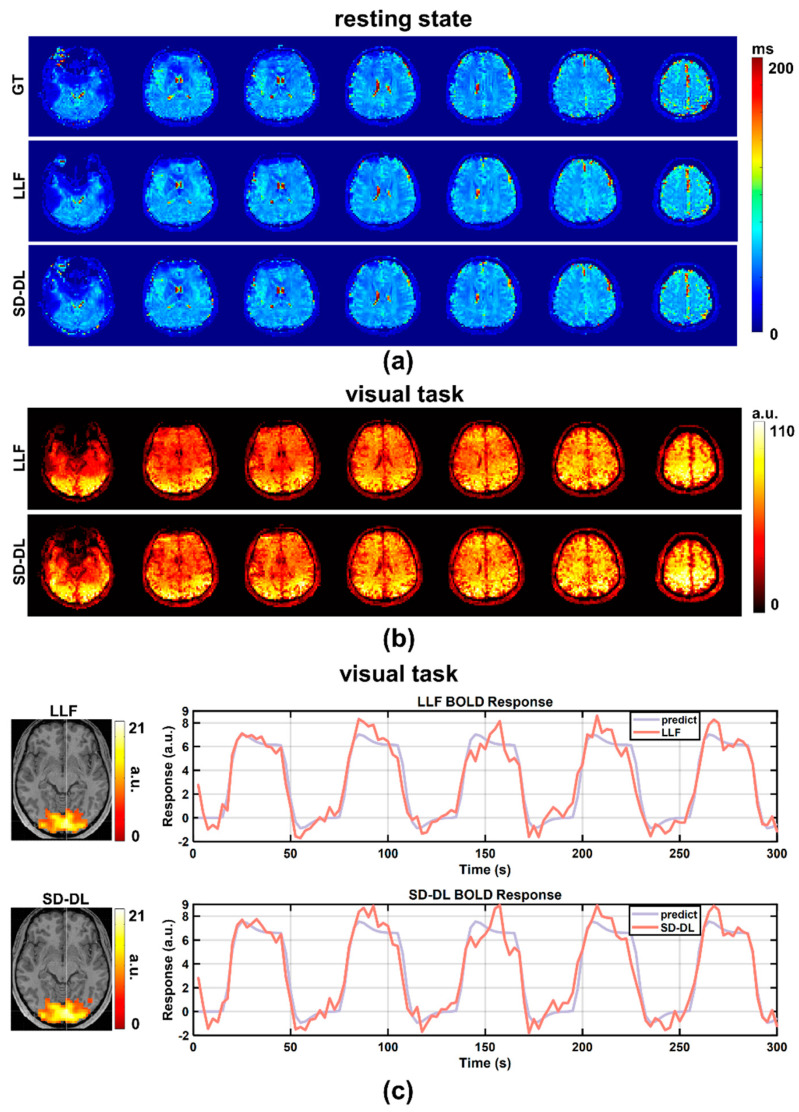
Comparison between LLF and SD-DL in *T*_2_* maps, tSNR maps, activation maps, and BOLD responses based on in-house fMRI data. (**a**) *T*_2_* maps estimated by LLF and SD-DL and the corresponding ground truth. (**b**) The tSNR maps of *T*_2_* time series estimated by LLF and SD-DL. (**c**) Activation maps (T-value maps) and BOLD responses. *p* < 0.0001. BOLD response curves were fitted with predicted responses from the maximum T-value voxel.

An individual subject’s resting-state *T*_2_* maps, derived from the in-house data, are shown in Figure 4a, with a *T*_2_* range of 0–200 ms. After averaging the 4D LLF and SD-DL-fitting resting-state *T*_2_* maps in the time dimension, 3D LLF and SD-DL-fitting resting-state *T*_2_* maps were obtained. In contrast to LLF, SD-DL was able to eliminate the noise in the *T*_2_* maps while preserving the textural features. tSNR maps of resting-state data are shown in Figure 4b. Compared to S_LLF_ (42.96), the whole brain tSNR of S_SD-DL_ (46.11) was 7.33% higher. Activation maps (T-value maps) and BOLD curves of S_LLF_ and S_SD-DL_ are displayed in Figure 4c. The activation slice with the greatest T-value voxel is displayed on the left side of Figure 4c. Compared to S_LLF_, S_SD-DL_ exhibited a higher mean T-value. The voxel with the highest T-value yielded the BOLD response curve on the right. For both LLF and SD-DL, the BOLD response curves were in line with the expected curves. The SD-DL result showed a larger amplitude fluctuation (i.e., higher sensitivity) compared to that for the LLF method.

### 3.3. Results of rt-ME-fMRI Dataset

#### 3.3.1. tSNR

**Figure 5:**
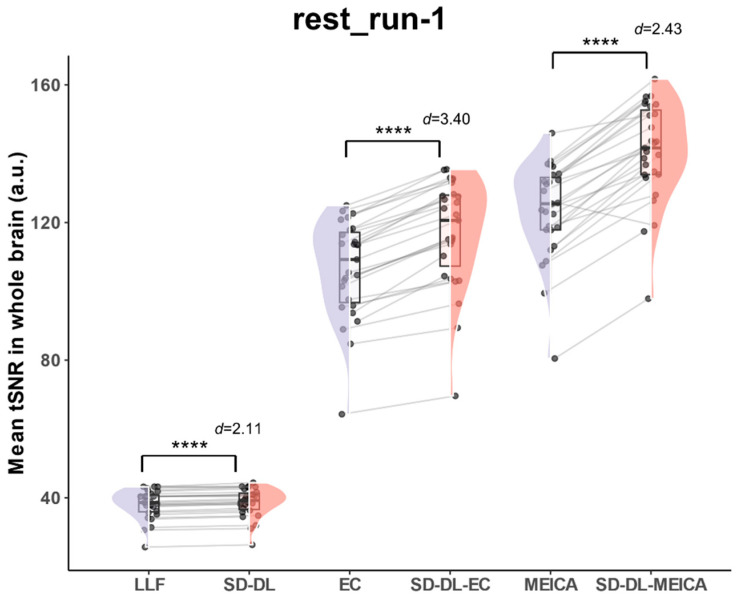
Comparison of whole-brain mean tSNR distribution of S_LLF_, S_SD-DL_, S_EC_, S_SD-DL-EC_, S_MEICA_, and S_SD-DL-MEICA_ in rt-ME-fMRI dataset. **** indicates *p* < 0.0001. *d* indicates Cohen’s *d*. Purple color indicates results using the LLF and orange-red color indicates results using the SD-DL.

With the goal of eliminating individual bias and providing a more quantitative view than the individual subject tSNR (Figure 4b), whole-brain mean tSNR was calculated directly from the 4D preprocessed time series without spatial smoothing using rest_run-1 data in the rt-ME-fMRI dataset. Figure 5 illustrates the whole-brain mean tSNR distribution of S_LLF_, S_SD-DL_, S_EC_, S_SD-DL-EC_, S_MEICA_, and S_SD-DL-MEICA_. In all the time series, the whole-brain mean tSNR of S_SD-DL_ was higher than that of S_LLF_. The whole-brain mean tSNR for S_SD-DL_ (38.68) was 1.47% higher than that for S_LLF_ (38.12), with *p* < 0.0001 and *d* = 2.11, and that for S_SD-DL-EC_ (116.9) was 9.77% higher than S_EC_ (106.5), with *p* < 0.0001 and *d* = 3.40, while the whole-brain mean tSNR for S_SD-DL-MEICA_ (140.6) exceeded that for S_MEICA_ (123.5) by 13.85%, with *p* < 0.0001 and *d* = 2.43.

#### 3.3.2. Percentage Signal Change

**Figure 6:**
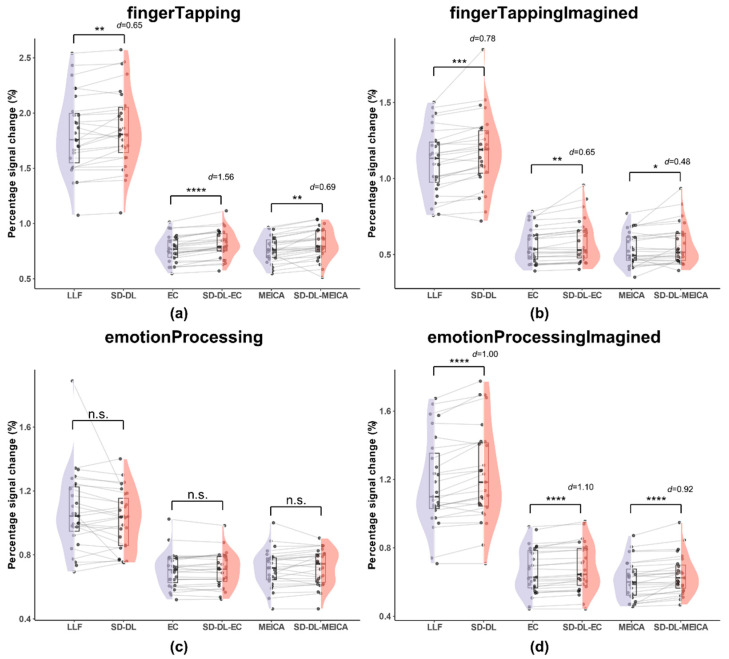
Comparison of percentage signal change within the UFWE of S_LLF_, S_SD-DL_, S_EC_, S_SD-DL-EC_, S_MEICA_, and S_SD-DL-MEICA_. The plots (from top to bottom) correspond to the following: (**a**) fingerTapping, (**b**) fingerTappingImagined, (**c**) emotionProcessing, and (**d**) emotionProcessingImagined. n.s., *, **, ***, and **** indicate no significance, *p* < 0.05, 0.01, 0.001, and 0.0001, respectively. Purple color indicates results using the LLF and orange-red color indicates results using the SD-DL.

The whole-brain PSC was calculated for each subject based on the fingerTapping task according to Equation (4). To quantify BOLD sensitivity equitably, the mean PSC distributions of all the time series within the UFWE were calculated. Figure 6 illustrates the mean PSC distributions within the UFWE of S_LLF_, S_SD-DL_, S_EC_, S_SD-DL-EC_, S_MEICA_, and S_SD-DL-MEICA_.

As shown in Figure 6, the *T*_2_* time series exhibited higher PSCs, while the mean PSCs of S_LLF_ and S_SD-DL_ were much higher than those of the other four methods. Specifically, for the fingerTapping task (Figure 6a), S_SD-DL_ (1.863) had a 2.59% higher PSC than S_LLF_ (1.816), with *p* < 0.01 and *d* = 0.65, while that of S_SD-DL-EC_ (0.8117) exceeded that of S_EC_ (0.7682) by 5.66%, with *p* < 0.0001 and *d* = 1.56, and that of S_SD-DL-MEICA_ (0.8142) exceeded that of S_MEICA_ (0.7677) by 6.06%, with *p* < 0.01 and *d* = 0.69. For the fingerTappingImagined task (Figure 6b), S_SD-DL_ (1.173) had a 5.01% higher PSC than S_LLF_ (1.117), with *p* < 0.001 and *d* = 0.78, while that of S_SD-DL-EC_ (0.586) exceeded that of S_EC_ (0.558) by 5.02%, with *p* < 0.01 and *d* = 0.65, and that of S_SD-DL-MEICA_ (0.566) exceeded that of S_MEICA_ (0.535) by 5.79%, with *p* < 0.05 and *d* = 0.48. For the emotionProcessing task (Figure 6c), the *t*-test of PSCs between S_LLF_ (1.069) and S_SD-DL_ (1.020), S_EC_ (0.704) and S_SD-DL-EC_ (0.706), S_MEICA_ (0.713) and S_SD-DL-MEICA_ (0.722) were not significantly different. For the emotionProcessingImagined task (Figure 6d), S_SD-DL_ (1.230) had a 4.06% higher PSC than S_LLF_ (1.182), with *p* < 0.0001 and *d* = 1.00, while that of S_SD-DL-EC_ (0.683) exceeded that of S_EC_ (0.666) by 2.55%, with *p* < 0.0001 and *d* = 1.10, and that of S_SD-DL-MEICA_ (0.642) exceeded that of S_MEICA_ (0.614) by 4.56%, with *p* < 0.0001 and *d* = 0.92.

#### 3.3.3. T-Values

**Figure 7:**
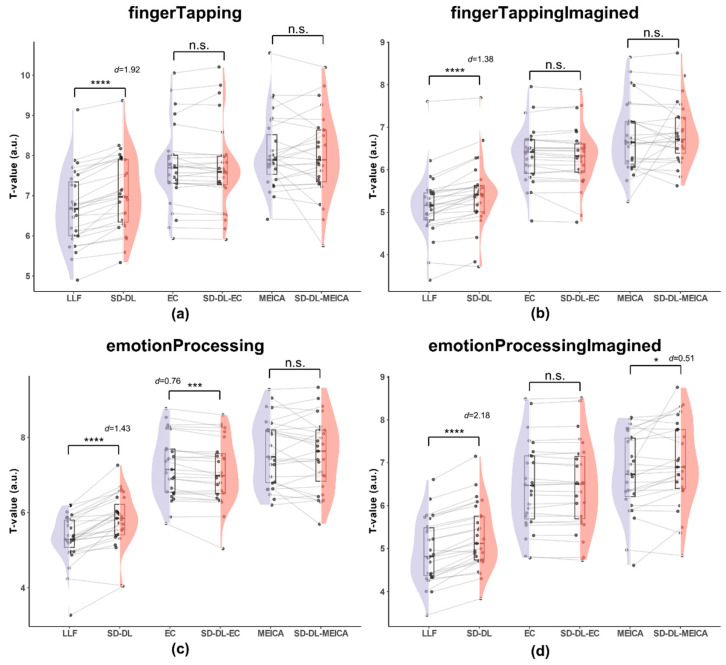
Comparison of the T-values within the UFWE for S_LLF_, S_SD-DL_, S_EC_, S_SD-DL-EC_, S_MEICA_, and S_SD-DL-MEICA_. The plots (from top to bottom) correspond to the following: (**a**) fingerTapping, (**b**) fingerTappingImagined, (**c**) emotionProcessing, and (**d**) emotionProcessingImagined. n.s., *, ***, and **** indicate no significance, *p* < 0.05, 0.001, and 0.0001, respectively. Purple color indicates results using the LLF and orange-red color indicates results using the SD-DL.

T-values were calculated after GLM analysis within the UFWE using fingerTapping data in rt-ME-fMRI. Figure 7 illustrates the distribution of mean T-values (over all participants) of S_LLF_, S_SD-DL_, S_EC_, S_SD-DL-EC_, S_MEICA_, and S_SD-DL-MEICA_ within the UFWE.

As shown in Figure 7, for the fingerTapping task (Figure 7a), the average of mean T-value of S_SD-DL_ (7.065) was 5.62% higher than that of S_LLF_ (6.689), with *p* < 0.0001 and *d* = 1.92, while the *t*-test of the averages of the mean T-values between S_EC_ (7.724) and S_SD-DL-EC_ (7.711), S_MEICA_ (8.063), and S_SD-DL-MEICA_ (8.000) were not significantly different. For the fingerTappingImagined task (Figure 7b), the average of the mean T-values of S_SD-DL_ (5.516) was 6.90% higher than that of S_LLF_ (5.160), with *p* < 0.0001 and *d* = 1.38, while, according to *t*-test, the averages of the mean T-values between S_EC_ (6.349) and S_SD-DL-EC_ (6.313), S_MEICA_ (6.778), and S_SD-DL-MEICA_ (6.861) were not significantly different. For the emotionProcessing task (Figure 7c), the average of the mean T-values of S_SD-DL_ (5.789) was 8.88% higher than that of S_LLF_ (5.317), with *p* < 0.0001 and *d* = 1.43, and S_EC_ (7.221) had a 1.66% higher average of mean T-values than S_SD-DL = EC_ (7.104), with *p* < 0.001 and *d* = 0.76, while, according to *t*-test, the averages of the mean T-values between S_MEICA_ (7.622) and S_SD-DL-MEICA_ (7.553) were not significantly different. For the emotionProcessingImagined task (Figure 7d), the average of the mean T-values of S_SD-DL_ (5.265) was 6.62% higher than that of S_LLF_ (4.938), with *p* < 0.0001 and *d* = 2.18, and S_SD-DL-MEICA_ (6.974) exceeded S_MEICA_ (6.754) by 3.26%, with *p* < 0.05 and *d* = 0.51, while the results of the *t*-test of the averages of the mean T-values between S_EC_ (6.459) and S_SD-DL-EC_ (6.454) were not significantly different.

#### 3.3.4. Functional Contrast

**Figure 8:**
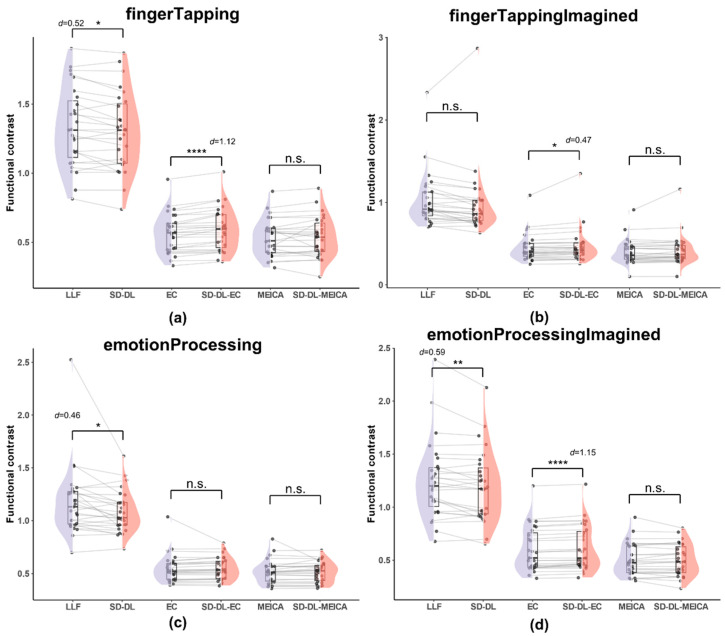
Comparison of functional contrast distribution within the UFWE for S_LLF_, S_SD-DL_, S_EC_, S_SD-DL-EC_, S_MEICA_, and S_SD-DL-MEICA_. The plots (from top to bottom) correspond to the following: (**a**) fingerTapping, (**b**) fingerTappingImagined, (**c**) emotionProcessing, and (**d**) emotionProcessingImagined. n.s., *, **, and **** indicates no significance, *p* < 0.05, 0.01, and 0.0001, respectively. Purple color indicates results using the LLF and orange-red color indicates results using the SD-DL.

FC was calculated within the UFWE ROI using fingerTapping data in rt-me-fMRI. Figure 8 illustrates the distribution of FC of S_LLF_, S_SD-DL_, S_EC_, S_SD-DL-EC_, S_MEICA_, and S_SD-DL-MEICA_ within the UFWE.

As shown in Figure 8, both S_LLF_ and S_SD-DL_ demonstrated a higher average FC than S_EC_, S_SD-DL-EC_, S_MEICA_, and S_SD-DL-MEICA_. Specifically, for the fingerTapping task (Figure 8a), S_LLF_ (1.330) exceeded S_SD-DL_ (1.300) by 2.31%, with *p* < 0.05 and *d* = 0.52; S_SD-DL-EC_ (0.591) exceeded S_EC_ (0.569) by 3.86%, with *p* < 0.0001 and *d* = 1.12. Meanwhile, the *t*-test results for S_MEICA_ (0.532) and S_SD-DL-MEICA_ (0.538) were not significantly different. For the fingerTappingImagined task (Figure 8b), S_SD-DL-EC_ (0.472) exceeded S_EC_ (0.448) by 5.36%, with *p* < 0.05 and *d* = 0.47, while the *t*-test results for S_LLF_ (1.023) and S_SD-DL_ (0.982), S_MEICA_ (0.401), and S_SD-DL-MEICA_ (0.415) were not significantly different. For the emotionProcessing task (Figure 8c), S_LLF_ (1.175) exceeded S_SD-DL_ (1.086) by 8.20%, with *p* < 0.05 and *d* = 0.46. The *t*-test results for S_EC_ (0.549) and S_SD-DL-EC_ (0.547), S_MEICA_ (0.517), and S_SD-DL-MEICA_ (0.515) were not significantly different. For the emotionProcessingImagined task (Figure 8d), S_LLF_ (1.247) exceeded S_SD-DL_ (1.200) by 3.92%, with *p* < 0.01 and *d* = 0.59; S_SD-DL-EC_ (0.614) exceeded S_EC_ (0.600) by 2.33%, with *p* < 0.0001 and *d* = 1.15. The *t*-test results between S_MEICA_ (0.505) and S_SD-DL-MEICA_ (0.503) were not significantly different.

## 4. Discussion

In this study, we have presented an SD-DL method for *T*_2_* mapping from multi-echo functional magnetic resonance imaging (ME-fMRI) datasets. The efficacy of SD-DL was examined. Human brain templates derived from publicly available datasets were used to synthesize training data. To enhance the neural network model’s ability to recognize noise in real ME-fMRI data, a controlled level of noise was introduced to the synthetic data. The BOLD sensitivity and image quality between SD-DL and traditional voxel-by-voxel LLF methods were compared.

Theoretically, LLF represents the gold standard for *T*_2_* estimation in fMRI devoid of noise contamination. However, simulation experiments have revealed that the MSE of LLF is significantly greater than that observed with the SD-DL when performing *T*_2_* mapping based on signals that added noises. This disparity suggests that SD-DL exhibits superior noise resilience over LLF, thereby enabling more effective handling of fMRI datasets characterized by diverse noise profiles. This enhanced noise robustness of SD-DL is further corroborated by a noticeable improvement in tSNR.

Regarding BOLD sensitivity, the *T*_2_* time series exhibits higher PSC and FC values compared to EC and MEICA, indicating that the *T*_2_*-based time series possesses higher sensitivity. Notably, the SD-DL *T*_2_* time series achieves higher PSC and comparable FC levels to those derived from LLF, implying an augmented sensitivity through SD-DL application. Furthermore, SD-DL-EC outperforms EC in both PSC and FC, and SD-DL-MEICA outperforms MEICA in PSC and displays a similar level of FC, suggesting SD-DL is capable of improving the performance of EC and MEICA. Additionally, SD-DL improves the mean T-value compared to LLF, indicating enhanced BOLD sensitivity without compromising specificity. Fundamentally, the BOLD effect arises from neuronal activity-induced fluctuations in the local magnetic field, subsequently altering the fMRI signal. Given that *T*_2_* is more responsive to such magnetic field changes, it inherently has a higher BOLD sensitivity. Nonetheless, in routine fMRI investigations, *T*_2_* mapping is challenged by noise contamination, limiting its broad applicability. SD-DL, on the other hand, can effectively mitigate the noise interference, consequently improving the BOLD sensitivity.

While *T*_2_* time series, S_EC_, and S_MEICA_ have distinct advantages or disadvantages in ME-fMRI across various indicators, SD-DL consistently shows superior performance in most indicators for different types of time series. Moreover, even if the data have different matrix sizes, resolutions, or sets of echo times, simulation samples can be generated and a neural network can be trained through the SD-DL framework. Although deep learning is sensitive to the training set and training method, we use synthetic data to minimize its dependence on real data and the risk of overfitting, and at the same time, we use a simpler network structure involving fewer hyperparameters, and these manipulations make SD-DL higher applicable and reproducible. Moreover, the introduction of a known level of noise to the synthetic samples enables the generation of data that more closely resemble actual fMRI data. Additionally, U-net possesses a large receptive field, allowing it to capture a lot of information in the image, while jump connections ensure that the convolutional neural network can maximize the preservation of the original information during deep processing and effectively realize the denoising function, which further enhances the accuracy and reliability of *T*_2_* mapping. In contrast, the LLF method is unable to account for the presence of noise when performing *T*_2_* mapping, which may result in amplified fitting errors due to numerical uncertainties.

Nevertheless, our proposed method for ME-fMRI has some limitations. The noise level introduced to SD-DL in the generation of simulation samples is an a priori knowledge that may result in suboptimal model performance if the added noise level is significantly different from the true noise level. The SD-DL *T*_2_* time series may not significantly improve tSNR due to the limited acquisition of three echoes, hindering high-accuracy *T*_2_* map reconstruction. Future studies should explore methods for obtaining more reliable *T*_2_* quantification in MR images under various means of accelerating imaging, potentially through the introduction of novel *T*_2_* quantitative imaging sequences [43].

Real-time fMRI-based studies have higher BOLD sensitivity requirements, and increasing the whole-brain mean tSNR is beneficial for real-time brain wide connectivity measurements [21]. Compared to that derived from the LLF method, the SD-DL-fitted *T*_2_* map has higher BOLD sensitivity and could be developed for adaptive paradigms and neurofeedback research.

The quality of synthetic data plays a crucial role in *T*_2_* map reconstruction. More precise modeling of non-ideal factors such as head movements in training data synthesis can enhance the generation of higher-quality *T*_2_* maps. This work highlights that SD-DL effectively reduces data noise while maintaining sensitivity similar to the LLF method, offering a new perspective for accurate *T*_2_* fitting in ME-fMRI data.

SD-DL could be applied to *T*_2_* mapping based on ME-fMRI data by adhering to the following steps: Firstly, generate synthetic samples using *M_0_* and *T*_2_* parameter templates based on the parameters of real-world data and the training model. Then, feed the preprocessed ME-fMRI data into the trained model to obtain *T*_2_* maps. Since SD-DL does not require the use of real-world training samples, it is possible to train the network so that it can complete *T*_2_* mapping on its own by only knowing the parameters of the acquired real-world data. Thus, SD-DL can be better integrated into the traditional multi-echo fMRI preprocessing process.

## 5. Conclusions

In this study, we proposed a ME-fMRI *T*_2_* mapping technique called SD-DL for accurate *T*_2_* map reconstruction from ME-fMRI data and compared the effects of LLF-fitted and SD-DL-fitted *T*_2_* maps on BOLD sensitivity. SD-DL utilizes deep learning to model the mapping relationship between multi-echo fMRI signals and *T*_2_* maps. By leveraging the powerful feature extraction and nonlinear mapping capabilities of deep neural networks, SD-DL is able to effectively capture the fundamental physical properties of the MRI acquisition process while accomplishing denoising, thereby improving robustness and generalizability. Compared to LLF-fitted *T*_2_* maps, SD-DL-fitted *T*_2_* maps have higher BOLD sensitivity, which helps to investigate the neural mechanisms of cognitive processes, brain diseases, and therapeutic interventions in more detail, meaning that the proposed method has a promising future in adaptive paradigms and neurofeedback research.

## Data Availability

The rt-ME-fMRI dataset (https://dataverse.nl/dataverse/rt-me-fmri (accessed on 15 August 2024)) [39] used in this study can be accessed via a data use agreement. The code is publicly available on https://github.com/yinghezhao/SD-DL (accessed on 15 August 2024).
